# The effects of interval aerobic training on mesenchymal biomarker gene expression, the rate of tumor volume, and cachexia in mice with breast cancer

**DOI:** 10.22038/IJBMS.2019.39535.9375

**Published:** 2020-02

**Authors:** Samira Gholamian, Seyyed Reza Attarzadeh Hosseini, Amir Rashidlamir, Hamid Aghaalinejad

**Affiliations:** 1 Department of Exercise Physiology (Biochemistry and Metabolism), Faculty of Sports Sciences, Ferdowsi University of Mashhad, Mashhad, Iran; 2 Department of Physiology, Faculty of Sports Sciences, Ferdowsi University of Mashhad, Mashhad, Iran; 3 Department of Exercise Physiology, Faculty of Sports Sciences, Ferdowsi University of Mashhad, Mashhad, Iran; 4 Department of Sports Sciences, Faculty of Humanities, Tarbiat Modares University, Tehran, Iran

**Keywords:** Breast cancer, Cachexia, Interval aerobic training, TGF-β, Tumor Volume, Twist, Vimentin

## Abstract

**Objective(s):**

It seems that regular exercise can have inhibitory effects on the progression of breast cancer. This study, therefore, aimed to investigate the influences of interval aerobic training on mesenchymal biomarker gene expression, muscle cachexia, and tumor volume changes in mice with breast cancer.

**Materials and Methods:**

Thirty-two female Balb/c mice were allocated to four groups: Exercise Tumor Exercise, Rest Tumor Rest (Control), Rest Tumor Exercise, and Exercise Tumor Rest. Interval aerobic training was done 6 weeks before and 4 weeks after tumor induction. Weight test and inverted screen test were carried out as muscle function tests. Data were analyzed using one-way ANOVA and HSD *post hoc*.

**Results:**

The results showed a significant decrease in gene expressions of Twist, Vimentin, and TGF-β in Exercise Tumor Exercise group in comparison with the Control group (*P<*0.05). Remarkable reduction of the rate of tumor volume was also observed in two training groups (Rest Tumor Exercise, Exercise Tumor Exercise) compared with the control group. According to function tests’ results, muscle functions were diminished due to cancer, but interval aerobic training can keep muscles in a normally-functioning state in cancer (*P<*0.05).

**Conclusion:**

Considering final results, a period of interval aerobic training can be used not only as a prevention method, but also help cancer treatment and impede cachexia by tumor volume reduction, decrease mesenchymal biomarker gene expression, and increase muscle strength functions.

## Introduction

Breast cancer is a growing health problem worldwide (1). Metastasis, the main cause of cancer deaths, involves dynamic and multiple biological processes, proceeding of which requires cancer cells to be able to break away from adjacent cells. These detached cells target extracellular matrix and basement membrane, enter the bloodstream, and at last far tissues. They leave the blood then and get into mesenchyme tissue, where they reproduce and form a secondary tumor (2, 3). EMT (epithelial-mesenchymal transition) is a crucial point in the metastasis cascade. Several broad studies have shown the correlation between EMT and cancer progression due to metastasis (4). EMT is a process by which epithelial cells transform into mesenchymal cells, with much invasiveness and mobility. Epithelial cells first form an integrated complex; then they gain the ability to move as a result of losing adherence junction proteins and cytoskeleton elements, and perturbation of E-cadherin. Finally, abnormal expression of specific Cadherins leads to the mesenchymal conversion of these cells (5). The EMT process is regulated by a set of transcription factors such as TGF-β, Twist, and Vimentin (6). The Vimentin gene is located on chromosome 10p13. Vimentin is a 466-amino-acid structural protein that contributes to cell-matrix adhesion (7, 8).

Twist protein (helix-loop- helixld), 21-kDa transcription factor, has been found in solid human tumors such as some types of cancer, sarcoma, glioma tumor, neuroblastoma, and melanoma cancer (9). Upregulation of Twist and Vimentin, repression of E-cadherin, and induction of EMT process result in increased invasiveness and metastasis of cancerous cells (10). 

TGF-β (Transforming growth factor-beta) is a 25 kDa polypeptide. The TGF-β intracellular signaling pathway is facilitated by Smads, and finally, the signal is delivered to the nucleus to change the regulation of DNA transcription (11). TGF-β signaling is involved in growth inhibition of tumor cells and can act as a tumor suppressor during the initial stages of carcinogenesis. So TGF-β can be considered a potential inducer of EMT and a crucial factor in cancer progression (12, 13). The TGF-β pathway can induce the EMT procedure through dependent or independent-Smad pathways. This inducer and Tyrosine Kinase Receptor (RTK) ligands can make changes in gene expression via complicated signaling networks. The final result of these processes is up to the regulation of transcription suppressors like Snail, Slug, Vimentin, and Twist proteins. TGF-β leads EMT to use tumor mediators to activate anti-apoptosis signaling pathways (14).

Cachexia is a complex metabolic syndrome that causes muscle loss with or without body fat mass reduction. Muscle cachexia is correlated with loss of appetite, inflammation, insulin resistance, and an increase of proteolysis (15). Cancer cachexia can be considered one of the most severe muscle dystrophies and is related to the high rate of mortality (16). Many patients with advanced cancer often experience unintentional weight loss. Cancer cachexia normally occurs with loss of appetite and leads to loss of physical functions and delirium (17). In research, it is declared that overexpression of Twist in both mesenchymal cancerous cells and muscle cells can influence muscle mass (18). Physical practices seem to be a preventing mechanism of cachexia in cancer patients (19). It is suggested that endurance training will raise the antioxidant capacity of skeletal muscles, thereby keeping the muscle mass. Physical exercise can lower fat percentage, reduce obesity, and decrease low-grade systemic inflammation, which all play a role in cancer pathogenesis. Thus, physical training is even likely to prevent cancer (20). There are several studies concerning the effect of exercise on the EMT process, although few types of research have been done to show training consequences on mesenchymal biomarker gene expression in cancer. Zhang *et al.* research on mice with hepatic cancer indicated a significant decrease in mesenchymal biomarker gene expressions after mild swimming practices (8 min per day) for 9 weeks. With great emphasis on the nervous system, they declared that mild swimming training could raise dopamine secretion, consequently activate dopamine D2 receptor, which has anti-tumor activity; accordingly, the EMT process is suppressed, and tumor development is prevented (21). Some advantages of interval training for breast cancer patients are increased vital capacity of the lungs, increased muscle endurance, weight loss, and efficient cardiac metabolism (22).

Also, several studies have found that drug inhibition of Twist can cease cachexia and its related death. Hence, it is assumed that Twist inactivation not only can rehabilitate the muscle mass needed for natural life activities but also can increase the life expectancy of affected people. However, very little research has been done so far to recognize the cellular and molecular mechanism of aerobic exercise and how it can affect related signaling pathways to prevent cachexia and tumor growth. Moreover, many available drugs cannot treat cancer effectively due to several reasons like being uni-target, high toxicity, excessive price, and the possibility of relapse, tumor growth, and cell adhesion. As a result, aerobic exercises can be a functional preventing method, as well as an effective part of a cancer cure. The complication and extensive nature of the EMT process makes it a big challenge to study precisely. But science achievements and extreme speed of approaches can be encouraging to set a broad investigation of molecular details of the EMT process in mice with cancer. This research aimed to evaluate interval aerobic training influence on mesenchymal biomarker gene expressions, tumor volume, and muscle cachexia in mice with breast cancer.

## Materials and Methods

This survey is an experimental and basic study done as controlled laboratory research. All ethical principles were according to the principles of laboratory animal research of Pasteur Institute of Iran. Also, the research pattern and details of every stage were studied and confirmed by the Ethics Committee of Ferdowsi University of Mashhad (IR.MUM.FUM.REC.1397.038).

Thirty-two female Balb/c mice (3-5 weeks old, average weight of 17±1 g) were divided randomly into four groups of 8. The groups included 1) Exercise-Tumor-Exercise (ETE), 2) Rest-Tumor-Rest (RTR), 3) Rest-Tumor-Exercise (RTE), and 4) Exercise-Tumor-Rest (ETR). The RTR group had its usual lifestyle as a control group. The other three groups were exercised according to the training protocol shown in Table 1. ETR group had training practices 6 weeks before tumor induction, RTE group 4 weeks after, and ETE group 6 weeks before and 4 weeks after tumor injection. The training protocol in Table 1 is based on Ranjbar and colleagues protocol (23) (Table 1).

***Cell culture***

Tumor cells, obtained from the 4T-1 cell line, were injected subcutaneously. The new cancerous cell line, which was produced from mouse tumors autonomously, was metastatic in mouse models, so they could move to other organs like the lungs, liver, brain, and bones, and finally created breast cancer (24). The cell line 4T-1 was cultured in the T75 flask in DMEM/F-12 medium containing 15 mM HEPES, glutamine, 100 µg/ml penicillin, 100 µg/ml streptomycin, and 10% FBS. After filling 90% of the flask with the cells, the supernatant in the medium was decanted. The cells were detached from the bottom of the flask by using 0.025% trypsin, and after the process of rinsing with PBS and enzyme neutralization using 10% FBS, all the flask contents were emptied into Falcon tubes and centrifuged at 1200 rpm for 3 to 5 min. In the next step, the supernatant was removed, and the cell plate dissolved in a medium containing 10% FBS. After that, trypan blue and a hemocytometer were used to determine cell viability and cell count, respectively. One million cells were subcutaneously injected into the upper part of the thighs. Ten days later, the tumor was visible in the injection area. Tumor volume was measured in vertical and horizontal axes. The most extended dimension was considered the length (L) of the tumor, and the other dimension (at the right angle to the length) was considered as the width (W) of the tumor. Tumor volume was calculated using the Jones volume formula V= ½ (L2×W) (25). In all groups, the rate of tumor growth was determined by division of the 4^th^ week volume to 1^st^ week volume.

***Functional tests***

These tests, including weight and inverted screen tests, were done at the beginning of the experiment, at the end of the primary training period, and at the last session of training before post-mortem examinations.

***Inverted screen test***

The inverted screen test was performed using a square wired net. The net was inverted while a mice was put at the center. The mice could hang upside down for a while, which was recorded. If the mouse was able to hang more, it was taken after 60 sec. The standard time for this study was 1 min, but more than this time can be useful for some other experiments (26).

***Weight test***

The weight test was carried out by seven rings with different weights. A mouse was forced to hold a ring, placed on the bench, with its middle part of the tail. The time was recorded when the mice were lifted as soon as holding the ball with paws until the ring was raised from the bench surface. The standard time of this test was 3 sec. More weights were added until the mice were not able to lift the weights. Scores of each mouse were given based on the specific criteria of this test (26).

***Tumor Tissue Extraction:***

All affected mice of the four groups were anesthetized by intraperitoneal injection of Ketamine (90 mg/kg) and Xylazine (10 mg/kg) after 24 hr from the last training session. The tumor was extracted and weighed under sterilized conditions, then frozen in liquid nitrogen. Tissue specimens were kept at -70 ^°^C for the next steps.

***mRNA Expression by Real-Time-PCR***

Total RNA from tumor tissue was extracted using an RNeasy Mini Kit (Qiagen, Germany), and total RNA (1 microgram) and small RNA (2 micrograms) were first reversely transcribed into cDNA using Transcriptor first-strand cDNA synthesis kit (Takara, Japan). Quantitative real-time PCR (qPCR) was performed with the SYBR Green RT-PCR Master Mix kit (Catalog No. 13D25, amplicon, Denmark), using HGS primers set (RDG. ID: 733650, Sina gene, Iran) and ACTB (as housekeeping) primers set. All samples were normalized to internal controls, and the relative expression level was calculated using the 2^-∆∆Ct^ analysis method. 

cDNA synthesis was performed using a Qiagen cDNA synthesis kit according to the manufacturer’s instructions. PCR Real-Time was performed using a device (Stepone plus AB Applied Biosystems). PCR cycles were performed with the final mixture of 20 µl volume, in 1 cycle at 95 ^°^C for 30 sec, 40 cycles at 95 ^°^C for 5 sec, and 60 ^°^C for 30 sec, respectively. Amplified DNAs were confirmed by the melting curve at the end of each PCR cycle. Primers were designed by Runner Gene Primer Software (Table 2). 

***Statistical analysis***

After ensuring that the distribution of weight data was normal, the Kolmogorov Smirnov test was used. One-way ANOVA and Turkey’s *post hoc* test were used to determine the difference between the groups. Mann-Whitney U test and Wilcoxon test were used to assess the effects of variants in functional tests and variants pre- and post-tumor injection, respectively. All statistical analyses were performed using SPSS software version 22 and *P*<0.05 significance level.

## Results

The results of this examination indicated significant influence of cancer in gastrocnemius muscle weight. Also, it is shown that muscle mass balance was kept with the least changes in the ETE group.

Table 3 represents changes in bodyweight with or without tumor, and the ratio of gastrocnemius weight to body weight in mice. The results of t-student showed a significant increase of bodyweight with the tumor in ETR, RTE, and RTR groups because of cancer (*P*<0.05), while this increase was not significant in the ETE group (*P*>0.05). Moreover, remarkably decreased ratio of gastrocnemius weight to bodyweight was found in ETR, RTR, and ETE groups after 10 weeks of interval aerobic training (*P*<0.05).

***The effect of interval aerobic training on TGF-*** ***β*** ***gene expression in tumor tissue***

**Figure 1:**
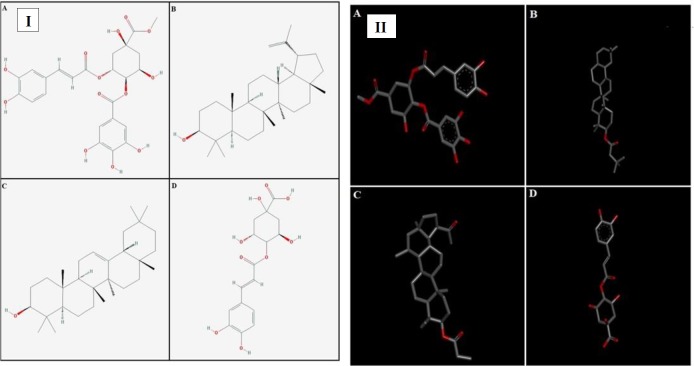
The changes of inter groups of TGF-β gene expression in compared with the control group

One-way ANOVA analysis resulted in similar conclusions as of the other two genes: notable reduction of TGF-β expression in ETE group compared with RTR(F=15/345, *P*=0.005), and no significant difference among ETR and RTE groups (*P*<0.05) (Figure 1).

***The effect of interval aerobic training on Vimentin gene expression in tumor tissue***

**Figure 2:**
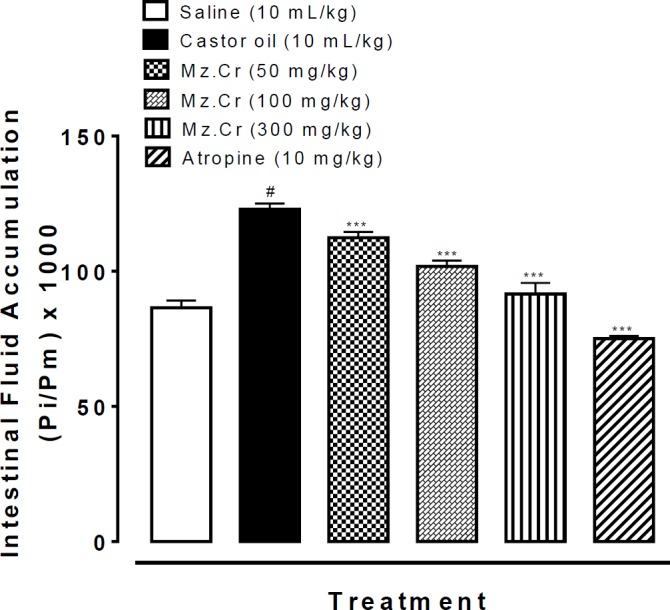
The changes of inter groups of Vimentin gene expression in compared with the control group

The results of one-way ANOVA analysis showed a remarkable difference of Vimentin gene expression in ETE and RTR groups (*P*<0.05) so that Vimentin expression had decreased significantly in tumor tissue of the ETE group (F=270/85, *P*=0.0001). However, any notable differences were not seen between RTE and ETR groups (*P*<0.05) (Figure 2). 

***The effect of interval aerobic training on Twist gene expression in tumor tissue***

**Figure 3:**
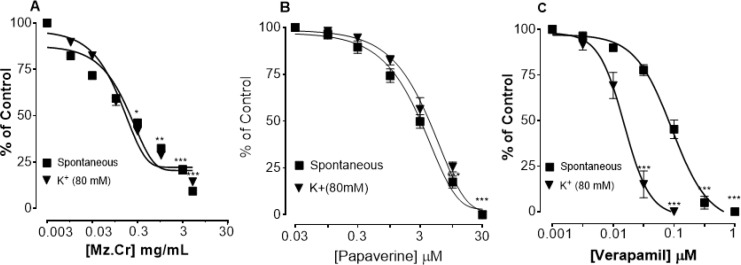
The changes of inter groups of Twist gene expression in compared with the control group

A significant decrease of Twist gene expression in the ETE group in comparison with the RTR group was recognized by doing One-way ANOVA analysis (*P*<0.05); so that Twis expression had decreased significantly in tumor tissue of ETE group (F=14/35, *P*=0.008). Here also, no remarkable change was seen between RTE and ETR groups (*P*<0.05) (Figure 3).

***The effect of interval aerobic training on tumor volume***

**Figure 4:**
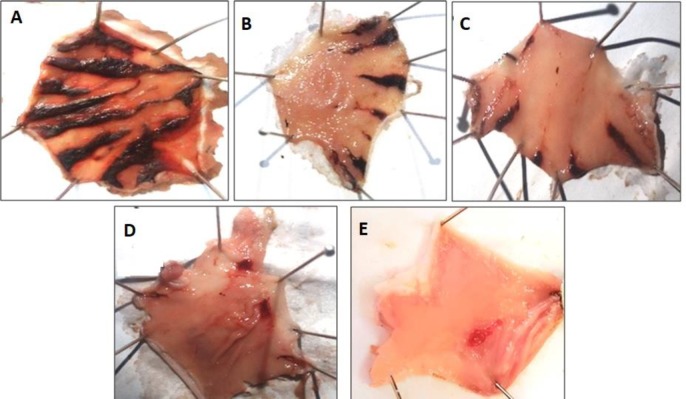
The Comparison of tumor volumes changes in first to fourth weeks after tumor induction in research groups

The ratio of tumor volume growth in the 4th week to the 1^st^ week showed a great decrease in RTE and ETE groups compared with the RTR group (F=23/81, *P*=0.0001) so that the ratio of tumor volume growth in ETE group was 4.79 after four weeks. While these ratios in RTE and ETR groups were 5.51 and 7. 5, respectively. The same ratio in the control group (RTR) was 8.92 after 4 weeks (Figure 4). 

According to the Wilcoxon signed-rank test, comparison of function tests before and after tumor induction in mice showed that cancer could cause a significant decrease in functional strength in ETR, RTR, and RTE groups (*P*<0.05), that is an indicative factor of cachexia in these groups after 4 weeks of tumor injection. Although some degrees of reduced functional strength were seen in the ETE group, too, this decrease was not significant (*P*>0.05). Hence it is deduced that doing interval aerobic training six weeks before and four weeks after tumor induction could maintain healthy functions of mouse models (Table 4).

## Discussion

Considering tumor volume growth and the decrease of TGF-β, Vimentin, and Twist expression in this study, it looks like the correlated chain, mediating between these regulation factors, can be known as a noble mechanism for the effect of interval aerobic training on breast cancer.

Some TGF-β signaling factors mutate during the transformation process of normal tissues to cancerous cells so that these cells will be resistant to TGF-β growth inhibition. These TGF-β-resistant cells and their surrounding stromal cells, called fibroblast cells, will divide uncontrollably. Therefore it can lead to high levels of TGF-β, immune system suppression, stimulation of angiogenesis and cell motility, increased interactions of tumor cells with the extracellular matrix, and increased tumor invasiveness. TGF-β can induce EMT in breast epithelial cells via both Smad-mediated and Smad-independent pathways. It can also cause the EMT process by increasing gene expressions of EMT-regulating transcription factors, such as Vimentin, Twist, Slug, and Snail. The duality of TGF-β was revealed by two different investigations; in one study, the lung metastasis was decreased by interruption of TGF-β signaling pathway, while in another research, increased TGF-β gene expression resulted in worsening the amelioration of disease (27).

Zhang *et al*. studied the effects of moderate swimming training (8 min per day) and high-intensity swimming (16 and 32 min per day) for nine weeks on TGF-β, Vimentin, and Twist gene expressions in C57 BL/6 mouse models with hepatic cancer. Their research revealed that moderate-intensity swimming could decrease TGF-β and mesenchymal biomarker gene expressions, while high-intensity swimming increases these biomarkers in tumor tissues of mice (21). It was indicated that moderate swimming activity increases dopamine (DA), and consequently activate dopamine receptors (DR2) (which have anti-tumor functions). Since ERK signaling pathway can increase both tumor invasiveness and TGF-β gene expression (28, 29), stimulating the DR2 signaling pathway with the help of physical training can result in cAMP decrease and suppression of ERK1/ERK2 activities (30), and consequently down-regulation of TGF-β and Smad3 in animal models (31). Therefore repressing Smad2, Smad3, and Smad4 nuclear translocation may regulate EMT-related TGF-β and suppress metastasis and tumor growth in lung cancer (27). These results align with our findings, although types of cancer, training protocols, and training periods were different in the two types of research (training periods were ten weeks in our study while it was nine weeks in Zhang *et al*. investigation).

A study evaluated the effects of endurance swimming training on changes of some cytokines like TGF-β in the pancreas of Balb-C mouse models with breast cancer. They found that swimming increases INF-γ and TNF-α cytokines; however, it decreases anti-inflammatory cytokines such as TGF-β and IL-10 in mice models with cancer (32). These results are also similar to our findings. Another study showed that a session of swimming for one hour could reduce TNF-α and TGF-β cytokines. According to their results, physical training can inhibit cancer cell divisions. They indicated that physical activity has anti-cancer influences, and can be prescribed as a cancer treatment (33). In Fairey *et al*. study, the exercise group was trained three times a week with an ergometer bicycle, although levels of neither inflammatory nor anti-inflammatory cytokines of blood cells (like TGF-β) showed any changes. As this study was performed on humans to evaluate cytokine levels that are released from blood cells (lymphocytes and macrophages) (34), blood samples were collected from all participants. In our survey, the subjects were Balb-C mice models, and tumor tissues were extracted, so it can explain the differences in the results. Also, Fairey *et al*. set three sessions of 15-35 min of training per week, whereas our training program was set to 5 times each week. It can be suggested that several sessions in a week and length of each session can be effective in changing cytokine levels, because we found a decrease in TGF-β level in tumor tissue, while there were no changes in released cytokines of blood cells in the other study.

Interval aerobic training can have prevention roles by having an effect on tumor volumes during the beginning weeks in mice. All mouse models were from the same race and got injected with equal numbers of cancer cells. So, different rates of tumor volume growth between RTE and ETE groups are believed to be due to the improvement of the immune system as a result of aerobic training. The first-week tumor volumes were smaller in mice that had done aerobic exercises compared with non-trained groups. As nursing methods and mouse models were exactly similar, and the only difference was doing interval aerobic training, it is possible to attribute the smaller tumor volume to aerobic training. Also, it was found in a study that running on the mouse wheels can decrease tumor volume in mouse models with HER2-positive breast cancer so that longer running results in smaller tumor volumes (35).

Several studies have reported the effective roles of physical activities in decreasing tumor volumes and speeding amelioration, although Colbert *et al*. declared that running on mice wheels increased tumor growth and reduced survival in breast cancer mouse models that lacked the P53-tumor suppressor gene (36). Several differences can be mentioned as the possible reasons for this contradiction; first, types of mice were not the same in the two kinds of research. Second, we started training activities for mice when they were five weeks old, while Colbert’s mice started training in their 10^th^ week. So it can be deduced that doing physical training at younger ages can be much more effective. Third, the period of our training protocol was exactly half (10 weeks in comparison with 20 weeks of Colbert’s research). It has been discovered in several other studies that angiogenic and inflammatory factors in a tumor microenvironment can be declined by physical activities, so it leads to fewer progressions of tumor growth (37, 38). 

Regarding all the investigations concerning the role of sports in cancer, it can be deduced that physical training is extremely helpful in both the prevention and amelioration of this malignant disease. However, it is noteworthy to mention that the practical role of training in breast and other cancer patients depends on different factors such as type and intensity of activities, number of sessions, and length of each session as well (33).

Molecular-biological changes resulting from doing exercises are widely unknown; so if successful studies are done about these changes, including different patterns of cytokine release in tumor tissues, it will be possible to employ efficient and well-timed physical activities to prevent and treat cancer.

Cachexia is associated with loss of muscle mass as well as muscle function. To determine cachexia, advanced devices must be used to measure the composition of the mouse body, which was a limitation of the current study. Since the nursing methods and the races of all mouse models were the same, the only difference was considered having cancer, which resulted in cachexia and weight loss. These conditions were accompanied by a decrease of TGF-β, Twist, and Vimentin gene expressions. Results of similar studies have attributed the changes to increased cytokine releases from tumor tissues and mainly macrophages and neutrophils (39). It has been recognized that TGF-β and Twist play an important role in cancer cachexia. Activin A proteins of the muscles lead to increased expression of Twist, and consequently MuRF1 and Atrogin1; so that muscular protein degradation and cachexia are the next occurrences (39). On the other hand, this survey showed the effects of cancer on body weight with and without tumor, the weight of gastrocnemius muscles, and muscle functions in mouse models. Interval aerobic training could mostly maintain the muscle mass and function, and partly prevent cachexia in mice suffering from cancer.

In addition, the tumor volume in the ETE group was shown to be smaller than the RTR group (which was aligned with previous investigations). A study indicated a significant decrease in tumor volumes in runner mice after three weeks of training. More lengths of running resulted in smaller tumors so that running more than 150 kilometers per week caused a remarkable reduction of tumor volume. In runner mouse models, the fat mass had an inverse relationship with tumor size. So decreased tumor volumes resulted in the increased weight of the fat mass. It represented fewer effects of cachexia on fat tissues in runner mice in contrast to non-running mouse models (35). However, cachexia is not a mono-factorial condition; most studies concerning cachexia declared that none of the possible factors could be solely considered as the main factor in cancer patients with or without weight loss (40). All these factors can weaken the functional and working states in patients, and have negative influences on chemotherapy efficiency (41).

Reduced tumor volume along with decreased expression of EMT biomarker gene can indicate notable effects of aerobic interval training on the EMT process in mice with breast cancer. It should be noted that few investigations have done so far about the evaluation of mesenchymal biomarker gene expressions and the rate of tumor volume (in breast cancer) as a result of physical activities, especially interval aerobic training. Therefore it will be quite difficult to interpret the results regarding the fact that there are a lot of effective factors in the EMT process or other possible factors may influence cancer. So the differences of tumor volumes between groups cannot be definitely considered due to variants of this research. It is suggested to investigate other related factors and mechanisms of the EMT process to be able to interpret the results more clearly.

**Table 1:** Training protocol before tumor injection and after tumor induction

**Training period**	**Speed(M/min)**	**Reiteration**	**Time(min)**	**Total Time(min)**	**Weeks**
**before injection of tumor**					
**Initiation stage**	10,12	-	-	10	3
**1** ^st^ ** and 2** ^nd^ ** Weeks**	15,20	20	2	40	5
**3** ^rd^ ** and 4** ^th^ ** weeks**	25,20	20	2	40	5
**5** ^th^ ** and 6** ^th^ ** weeks**	25,30	20	2	40	5
**Training after tumor induction**					
**7** ^th^ ** week**	25,30	15	2	30	5
**8** ^th^ ** week**	25,20	15	2	30	5
**9** ^th^ ** week**	15,20	15	2	30	5
**10** ^th^ ** week**	10,15	15	2	30	5

**Table 2:** Primer specification for cDNA synthesis

**Genes**	**Forward sequence**	**Reverse sequence**
Vimentin	ACATCATACGGCTGCGAGAG	GACTTGCTGTTCCTGAATCTGG
Twist	AGCAAAGCCTTCTCCGTCTG	CCTCCTCTGGAAACAATGACATC
TGF-β	TGGAGTTGGACGGCAGTG	TGGAGTTTGTTATCTTTGCTGTCAC
ACTB	GGCTGTATTCCCCTCCATCG	CCAGTTGGTAACAATGCCATGT

**Table 3:** The changes of bodyweight with or without tumor, and the ratio of gastrocnemius weight to body weight in groups

**Variable features**	**ETE**	**ETR**	**RTE**	**RTR**
**body weight without tumor (g)**	18/53±0/57	18/59±0/62	18/64±0/60	18/35±0/09
**body weight with tumor (g)**	19/83±0/67	20/47±0/34	20/67±0/28	21/31±0/43
**rate of gastrocnemius muscle weight to body weight**	0/043±0/12	0/020±0/09	0/030±0/14	0/018±0/10

ETE:Exercise-Tumor-Exercise, ETR:Exercise-Tumor-Rest, RTE:Rest-Tumor-Exercise and RTR: Rest-Tumor-Rest

RTR: Rest-Tumor-Rest, ETR:Exercise-Tumor-Rest, RTE:Rest-Tumor-Exercise and ETE:Exercise-Tumor-Exercise

RTR: Rest-Tumor-Rest, ETR:Exercise-Tumor-Rest, RTE:Rest-Tumor-Exercise and ETE:Exercise-Tumor-Exercise

RTR: Rest-Tumor-Rest, ETR:Exercise-Tumor-Rest, RTE:Rest-Tumor-Exercise and ETE:Exercise-Tumor-Exercise

RTR: Rest-Tumor-Rest, ETR:Exercise-Tumor-Rest, RTE:Rest-Tumor-Exercise and ETE:Exercise-Tumor-Exercise

**Table 4:** The comparison of the weight test and inverted screen test results before and after tumor induction in research groups

**TEST**		**Weight test**		**Inverted ** **screen test**	
**Group**	**Stage**	**Mean**	**Sig**	**Mean**	**Sig**
**RTR**	Pre	15±1/94	*P<0.*05*	4/20±0/421	*P<0.*05*
	Post	11/2±1/47		3±0/527	
**ETE**	Pre	15/7±1/8	*P*>0.05	4/70±0/483	*P*>0.05
	Post	15/30±1/5		4/53±0/427	
**ETR**	Pre	15/50±2/36	P<0.05*	4/60±0/51	*P*<0.05*
	Post	13/30±2/62		3/20±0/48	
**RTE**	Pre	15/22±1/92	*P*<0.05*	4/20±0/63	*P*<0.05*
	Post	13/45±1/81		4±0/53	

RTR: Rest-Tumor-Rest, ETE:Exercise-Tumor-Exercise, ETR:Exercise-Tumor-Rest and RTE:Rest-Tumor-Exercise

## Conclusion

This study revealed that interval aerobic training could have both preventing effects and cooperative roles in breast cancer treatment, in addition to inhibiting cancer cachexia. It is supposed that a period of interval aerobic training can reduce the rate of tumor growth, decrease mesenchymal biomarker gene expressions, and increase muscle strength function. However, understanding the mechanisms of these effects will require more investigations.
